# Supplementary material for: N6-Isopentenyladenosine Impairs Mitochondrial Metabolism through Inhibition of EGFR Translocation on Mitochondria in Glioblastoma Cells
Source: Cancers (Basel). 2022 Dec 8;14(24):6044. doi: 10.3390/cancers14246044 (PMC9776489; doi:10.3390/cancers14246044)
Supplement: Supplementary file 1 [file cancers-14-06044-s001.zip › cancers-1993511-supplementary.pdf]

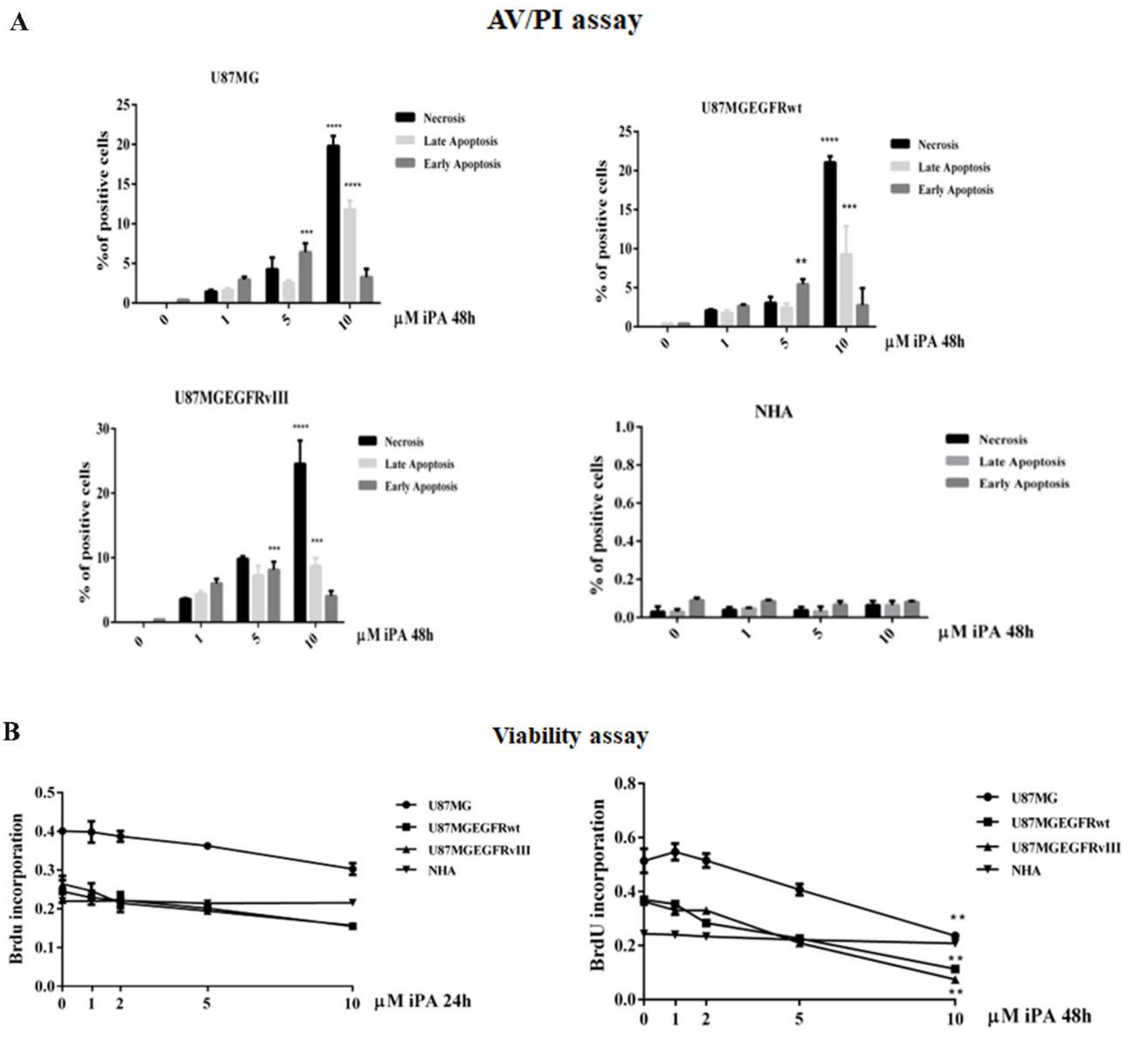

Figure S1: iPA reduces GBM cells proliferation rate and induces cell death. (A) GBM stabilized cell lines and normal control NHA cells were treated with 1, 5, and 10 µM of iPA for 24 h and 48h. Cell death was determined by flow cytometry followed by Annexin V/PI staining. Early Apoptosis: Annexin V-positive cells (AV+/PI−); Necrosis: PI-positive and Annexin V-negative cells (AV−/PI+); and Late apoptosis (AV+/PI+) (B) Antiproliferative effect of iPA on U87MG, U87-EGFRwt, U87-EGFRvIII GBM cell lines as detected by BrdU assay after 24 h and 48 h of treatment. ** $p < 0.01$, *** $p < 0.001$, **** $p < 0.0001$.

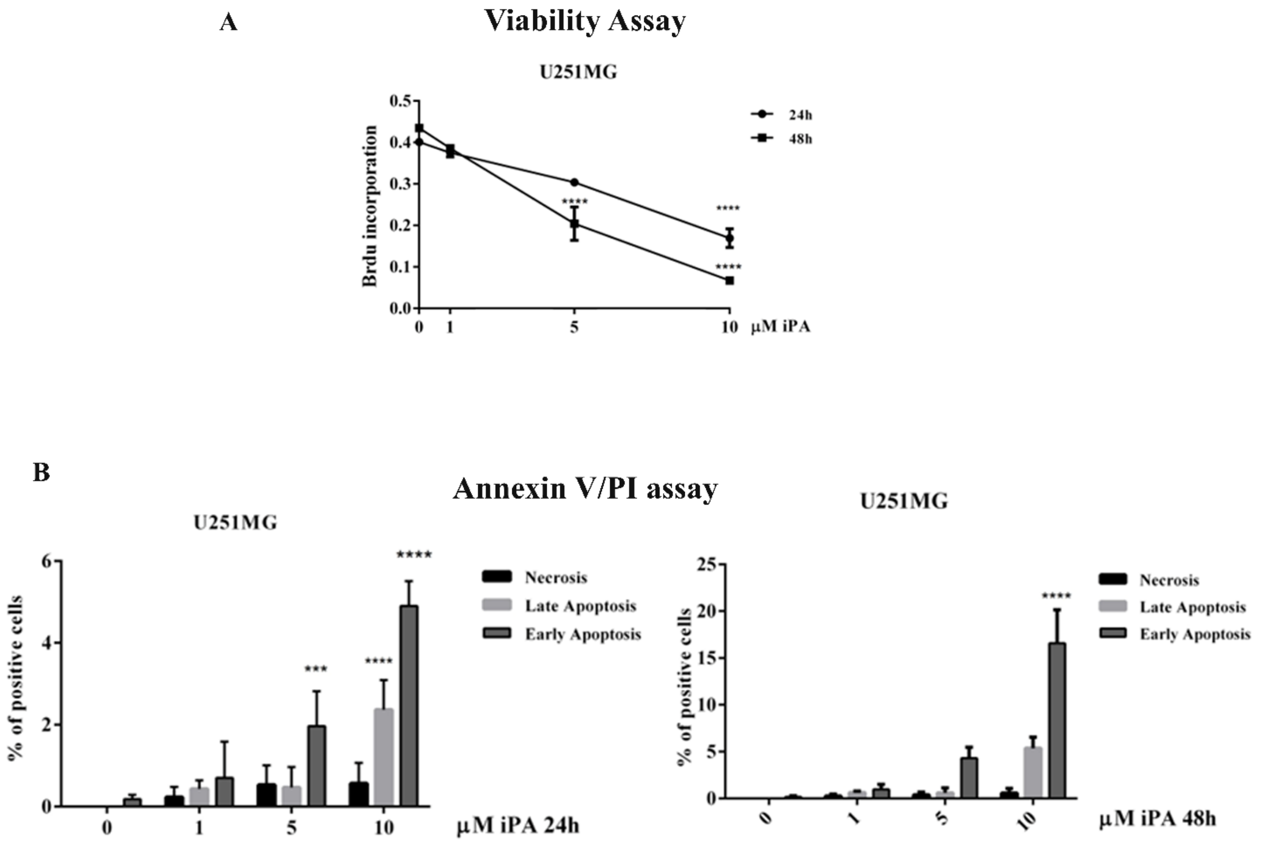

Figure S2: iPA reduces U251MG proliferation rate and induces cell death. (A) U251MG cells wew treated with 1, 5, and 10 µM of iPA for 24 h and 48h and its antiproliferative effect was detected by BrdU assay. (B) U251MG were treated with 1, 5, and 10 µM of iPA for 24 h and 48h. Cell death was determined by flow cytometry followed by Annexin V/PI staining. Early Apoptosis: Annexin V-positive cells (AV+/PI−); Necrosis: PI-positive and Annexin V-negative cells (AV−/PI+); and Late apoptosis (AV+/PI+). *** $p < 0.001$, **** $p < 0.0001$.

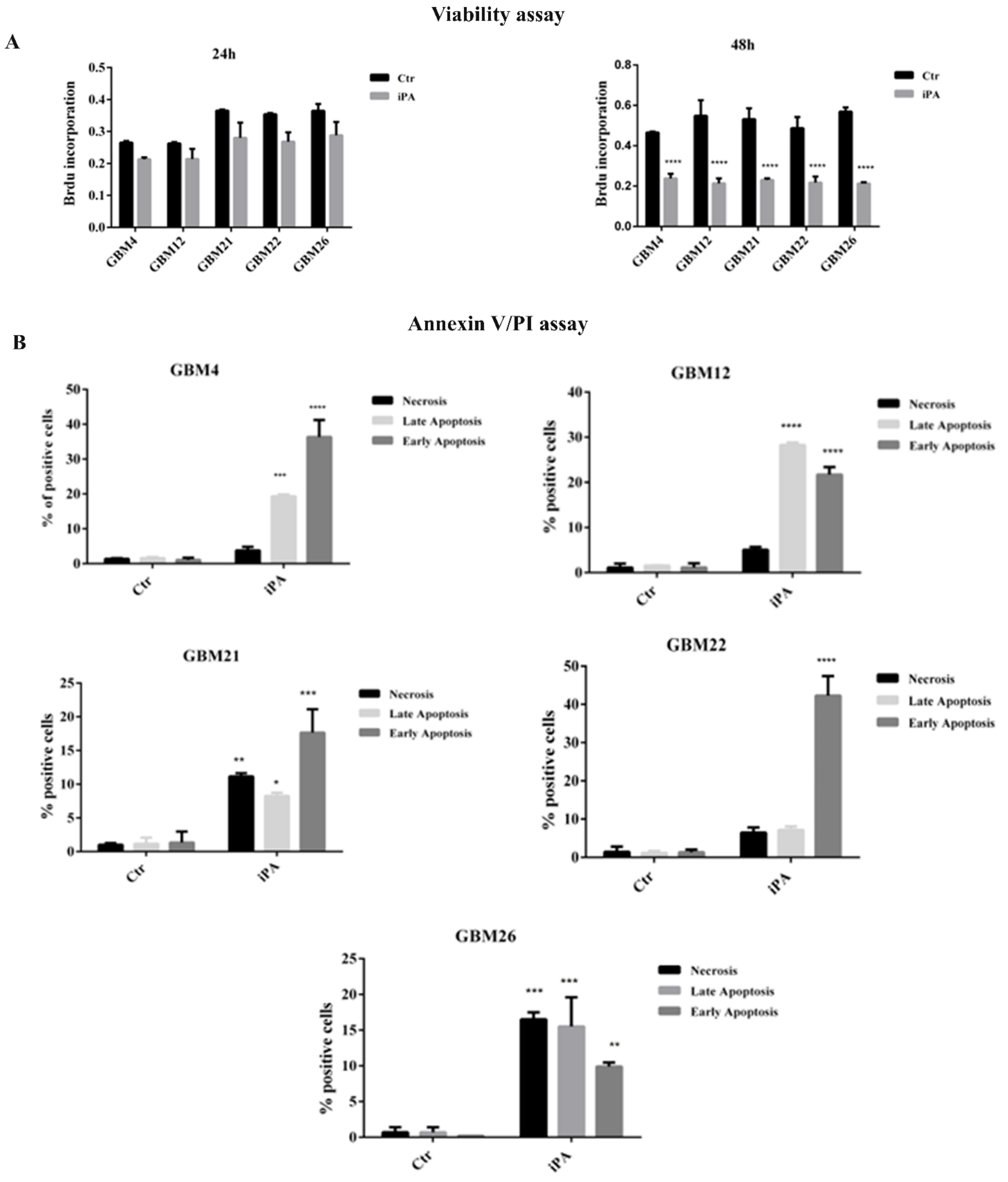

Figure S3: iPA reduces GBM primary cell lines proliferation rate and induces cell death. (A) Antiproliferative effect of iPA on GBM primary cell lines as detected by BrdU assay after 24 h and 48 h of treatment. (B) GBM primary cell lines were treated with iPA 10 µM for 48h. Cell death was determined by flow cytometry followed by Annexin V/PI staining. Early Apoptosis: Annexin V-

positive cells (AV+/PI−); Necrosis: PI-positive and Annexin V-negative cells (AV−/PI+); and Late apoptosis (AV+/PI+). * $p < 0.05$, ** $p < 0.01$, *** $p < 0.001$, **** $p < 0.0001$.

Supplementary Materials and Methods

BrdU assay

Proliferation assay was performed using the 5-bromo-2'-deoxyuridine ELISA kit (Roche, Basel, Switzerland), employed according to the manufacturer's protocol. The cells were seeded in 96-well plate, treated with iPA for 24 and 48 h and subsequently incubated for about two hours with 10µL of BrdU/well, added in the medium at a concentration of 100µM (BrdU Labeling Solution diluted 1: 100 in sterile medium). Subsequently, 100 µL of Kit-included FixDenant was added per well to fix the cells. Afterward, anti-BrdU-POD diluted according to instructions was added, cells were then washed with PBS 1x to remove the unbound antibody, and finally 100µl of Substrate Solution was added. The absorbance was measured at 450 nm using a Synergy HT Microplate Reader (BioTek Instruments Inc., Winooski, VT, USA).

Flow Citometry Analysis

The cells were grown at a density of $5x10^5$ cells/cm^2 in p60 dishes in supplemented DMEM and allowed to adhere for 24h before treatment. Assessment of apoptosis was conducted by human anti-annexin V staining and propidium iodide (PI) (Dojindo Molecular Technologies, MD, USA). Trypsin was used to harvest cells and PBS to wash them; cells were then resuspended in annexin V binding buffer (10 mM Hepes / NaOH, Ph 7; 140 mM NaCl; 2.5 mM CaCl2), stained with annexin V-FITC for 20 minutes at RT and then stained with PI at RT for 15 minutes in the dark. Cells were acquired by flow cytometer within 1 hour of staining. At least 10.000 events were collected and the data was analyzed by BD Accuri C6 software.
